# “Stabilize the Unstable”: Treatment Pathophysiology in Bleeding Trauma Patients, from the Field to the ICU. State of the Art

**DOI:** 10.3390/jpm13040667

**Published:** 2023-04-14

**Authors:** Roberto Bini, Francesco Virdis, Stefano Piero Bernardo Cioffi, Michele Altomare, Fabrizio Sammartano, Erika Borotto, Osvaldo Chiara, Stefania Cimbanassi

**Affiliations:** 1 Trauma Team ASST Niguarda, 20162 Milan, Italy; francesco.virdis@ospedaleniguarda.it (F.V.); stefanopiero.cioffi@ospedaleniguarda.it (S.P.B.C.); michele.altomare@ospedaleniguarda.it (M.A.); osvaldo.chiara@ospedaleniguarda.it (O.C.); stefania.cimbanassi@ospedaleniguarda.it (S.C.); 2 Trauma Center, San Carlo Borromeo ASST Santi Paolo e Carlo, 20162 Milan, Italy; fabrizio.sammartano@asst-santipaolocarlo.it; 3 Intensive Care Unit, Macchi Hospital, 21100 Varese, Italy; erikaborotto@gmail.com

The results of the Global Burden of Disease (GBD) study showed that, in 2019, 8% of deaths worldwide were trauma related. Moreover, the GBD reported the nonfatal sequelae (disabilities and handicaps) among trauma patient survivors, who are often healthy young individuals that will suffer lifelong disabilities as a result of the injuries sustained [1].

Thus, the management of haemodynamically unstable trauma patients continues to represent a challenge for clinicians who face a time-dependent condition in which critical injuries causing organ failure should be quickly recognized and controlled.

The quality of trauma care has improved remarkably worldwide since the beginning of the new century. Many countries developed efficient Trauma Systems based on the concept that pre-hospital personnel must recognize severe trauma in the field, support vital functions when needed, and finally centralize the patient to the facility of definitive care in the shortest possible timeframe. Specialized Trauma Centers have been implemented with the required resources to treat any type of injury 24/7 [2].

The US experience launched surgeons into full stewardship of trauma system development, while in Europe it is mainly led by anesthesiologists and emergency physicians [3].

The development of standardized courses in trauma care (i.e., Advanced Trauma Life Support ATLS, European Trauma Course ETC, Advanced Trauma Operative Management ATOM, Definitive Surgical Trauma Care DSTC) along with the implementation of centres dedicated to those patients and the use of advanced strategies and technologies (such as massive transfusion protocol, REBOA resuscitative endovascular balloon for aortic occlusion, TAG, ROTEM, etc.), and increased knowledge of physiological changes during shock and trauma, have helped reduce mortality and morbidity [4,5].

Trauma patients usually die because of brain injury or hemorrhagic shock; death from bleeding in trauma occurs within the first few hours after the hospital admission, and 60% of these deaths could potentially be avoided. Shock is a pathophysiological condition that occurs when oxygen delivery is unable to sustain aerobic metabolism in tissue [6].

Prolonged shock leads to cell death due to inadequate energy production, and an oxygen deficit arises when oxygen delivery falls below the levels necessary to support aerobic metabolism [6]. In this context, oxygen debt represents the degree of shock and correlates with death and complications related to shock, such as inflammation, acidosis, coagulopathy, and multiple organ failure [7].

Evidence suggests that hemorrhagic blood failure is initiated and propagated by blood loss and tissue damage, and it is defined by the presence of critically low tissue oxygen delivery, endotheliopathy, platelets dysfunction, and coagulopathy affecting clot formation. Plasma dilution, hypothermia, and acidosis can iatrogenically worsen coagulation [6].

Treatment of hemorrhagic blood failure requires that multiple components be addressed, including restoration of oxygen debt, treatment of both endothelial injury and coagulopathy, and stopping the bleeding [6].

Traumatic coagulopathy is a distinct and multilayered biochemical response to tissue injury and hemorrhagic shock [7].

Paracellular permeability leading to organ edema and organ failure is caused by a breakdown of endothelial cell–cell tight and adherent junctions that regulate the endothelial blood–organ barrier in various tissues [8].

Transfusion of packed red blood cells (PRBCs) primarily increases the oxygen-carrying capacity, cardiac output, and thus oxygen delivery to address the oxygen debt [8,9].

Plasma transfusion sustains cardiac output through intravascular volume expansion and coagulation factors to boost hemostasis; it may also provide endothelial protection by replacing important protective enzymes that can contribute to sealing the endothelial barrier, speed recovery of the glycocalyx, and rebalance thrombin generation through the provision of antithrombin [9,10].

Moreover, hemostasis is upheld by early platelet transfusion that improves outcomes in acutely bleeding trauma patients and cryoprecipitate that provide a source of fibrinogen, Factor VIII, and von Willebrand factor with an additional benefit to the endothelium [11].

Fresh whole blood and cold-stored whole blood can simultaneously address the three components of blood failure: oxygen debt, endotheliopathy, and coagulopathy. Their use is still debated in civil hospital environments and is more frequently considered in military settings [12].

Management of the bleeding trauma patient should start right on the field with well-defined strategies, the so-called “remote damage control resuscitation” (RDCR) [7].

If ABO-type-specific whole blood is not available, or if it is not feasible to accurately determine the ABO type of the donor and recipient, low-titer type O whole blood is ideal [13].

RDCR is typical of hostile environments but is comparable to prehospital rescue in Western countries (Figure 1).

Permissive hypotension, hemostatic resuscitation, and bleeding control (Tourniquets, pelvic Binder, REBOA) represent the main features of RDCR, together with ensuring adequate oxygenation through airway control and addressing the causes of obstructive shock [14].

The use of crystalloids should be minimized, while the infusion of tranexamic acid and calcium remains important. Where available, whole blood transfusion should be considered. Massive transfusion protocol includes Plasma, RBCs, and platelets in a 1:1:1 ratio or low-titer O whole blood (LTOWB) if available (more common in army and conflict areas).

Aside from early resuscitation, good communication and a clear handover in the admission trauma centre is essential to guarantee a proper definitive response; this includes the availability of a shock room, hybrid room, massive transfusion protocols, 24/7 operating theatres, etc.

Nowadays, modern Trauma Centers count on multidisciplinary teams, where each component follows standardized protocols to support vital functions, recognize the site and the severity of injuries and prioritize treatments.

The organizational model of the trauma system, prehospital setting, and trauma centers is the key to success in the treatment of unstable trauma patients. (Figure 2).

A bleeding patient’s access to the shock room represents the first of several steps in which a precise strategic sequence of events must be implemented to stop the hemorrhage. A damage control strategy should be initiated, in which anatomy meets physiology to stop the bleeding and promote homeostasis. (Figure 3).

Damage control principles can be summarized as controlling the hemorrhage, identifying injuries, controlling contaminations, and reconstruction. The abdomen is left open to prevent ACS and reduce the operative time.

On the way to the operating theatre, one of the most common challenges in the emergency department is deciding which cavity to explore first. Unless an obvious source of bleeding is suspected, first pack the pelvis while continuing the massive transfusion, then perform a damage control laparotomy, and then move to the chest, where a thoracotomy is required with 1.5 L, or 200 mL/h, of blood drained from the intercostal drain.

Table 1 reports the six reasons to perform abbreviated laparotomy (AL) identified by Moore when technical demands exceed the skills of the operating surgeon, irrespective of any coagulopathy [15].

The damage control resuscitation and surgery require the availability of a hybrid room which allows endovascular control to be performed before or after surgery to achieve hemostasis.

Patients should then be transferred to a dedicated ICU to restore body core temperature, coagulation, and hemodynamics to prepare them for the definitive surgical procedure [16].

A neuromuscular blockade is ideal to reduce the closure time in an open abdomen and use intraperitoneal dialysis with hypertonic saline [17].

Following a major inflammatory insult (trauma, sepsis, burns, acute pancreatitis, etc) there is a simultaneous inflammatory and immunosuppressive response. Early deaths from acute MOF are now rare due to early recognition of shock and rapid implementation of supportive care through the effective application of EBM and SOPs. Survivors may progress along two pathways: (a) patients returning to their immune homeostasis and achieve a rapid recovery; (b) patients deteriorate in the ICU with CCI (chronic critical illness) and develop chronic inflammation, suppression of adaptive immunity, ongoing protein catabolism with cachectic wasting, and recurrent nosocomial infections.

These patients often suffer from Persistent Inflammation, Immunosuppression, and Catabolism Syndrome (PICS), which describes a subgroup of patients with CCI who have experienced recurrent inflammatory insults, many of whom fail to achieve functional independence, and are discharged to LTACs (long-term acute care facilities (having an extremely poor quality of life leading to an indolent death [18].

Rapid decisions supported by scientific and clinical data, and simple to complex manoeuvres to stop or limit the bleeding and organ damage, are the keys to treating unstable trauma patients.

“Stabilize the unstable” patient is the cornerstone of trauma care and standardized protocols are necessary to achieve this result.

## Figures and Tables

**Figure 1:**
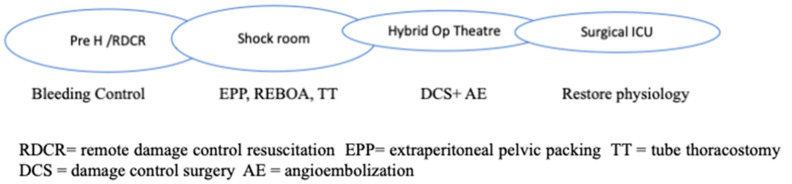
Chain of Trauma Survival.

**Figure 2:**
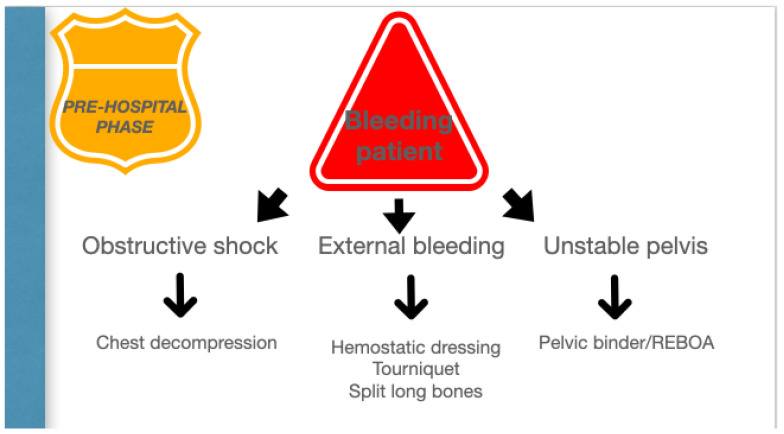
Pre-hospital phase.

**Figure 3:**
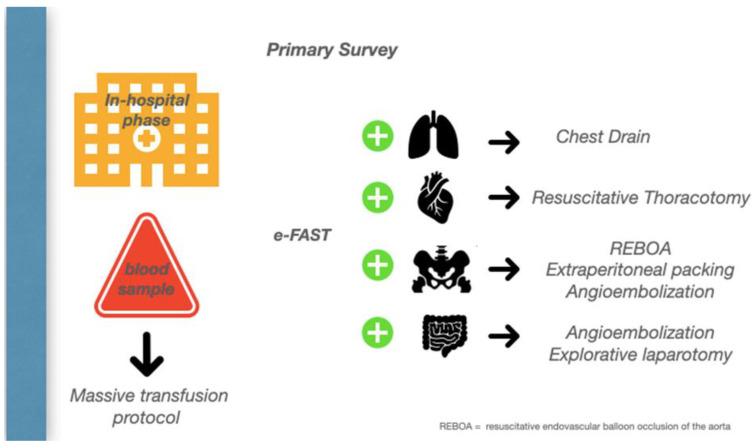
In-hospital Phase.

**Table 1:** Six reasons to perform abbreviated laparotomy [15].

**Coagulopathy (Vicious Circle)**	***Impossibility of Obtaining Correct Hemostasis***
**Vascular injuries difficult to access**	i.e., *difficult to reach the retrohepatic vessel*
**Time-consuming procedure**	i.e., *need for a pancreatoduodenectomy*
**Extra-abdominal injuries requiring AE**	i.e., *pelvic arterial blushing*
**Intra-abdominal hypertension**	*compromised abdominal wall closure*
**Need for abdominal organ revaluation**	i.e., *small bowel viability after a mesenteric injury*

## References

[1] Vos T., Lim S.S., Abbafati C., Abbas K.M., Abbasi M., Abbasifard M., Abbasi-Kangevari M., Abbastabar H., Abd-Allah F., Abdelalim A. (2020). Global burden of 369 diseases and injuries in 204 countries and territories, 1990–2019: A systematic analysis for the Global Burden of Disease Study 2019. Lancet.

[2] Chiara O., Cimbanassi S. (2003). Organized trauma care: Does volume matter and do trauma centers save lives?. Curr. Opin. Crit. Care.

[3] Peitzman A.B., Leppäniemi A., Kutcher M.E., Forsythe R.M., Rosengart M.R., Sperry J.L., Zuckerbraun B.S. (2015). Surgical Rescue: An Essential Component of Acute Care Surgery. Scand. J. Surg..

[4] Soreide K. (2009). Trauma and the acute care surgery model—Should it embrace or replace general surgery?. Scand. J. Trauma Resusc. Emerg. Med..

[5] King D.R. (2019). Initial Care of the Severely Injured Patient. N. Engl. J. Med..

[6] White N.J., Ward K.R., Pati S., Strandenes G., Cap A.P. (2017). Hemorrhagic blood failure: Oxygen debt, coagulopathy, and endothelial damage. J. Trauma Acute Care Surg..

[7] Jenkins D.H., Rappold J.F., Badloe J.F., Berséus O., Blackbourne C.L., Brohi K.H., Butler F.K., Cap L.A.P., Cohen M.J., Davenport R. (2014). Trauma hemostasis and oxygenation research position paper on remote damage control resuscitation: Definitions, current practice, and knowledge gaps. Shock.

[8] Thurston G., Rudge J.S., Ioffe E., Zhou H., Ross L., Croll S.D., Glazer N., Holash J., McDonald D.M., Yancopoulos G.D. (2000). Angiopoietin-1 protects the adult vasculature against plasma leakage. Nat. Med..

[9] Kozar R.A., Peng Z., Zhang R., Holcomb J.B., Pati S., Park P., Ko T.C., Paredes A. (2011). Plasma restoration of endothelial glycocalyx in a rodent model of hemorrhagic shock. Anesth. Analg..

[10] Cardenas J.C., Cap A.P., Swartz M.D., Huby M.D.P., Baer L.A., Matijevic N., Cotton B.A., Holcomb J.B., Wade C.E. (2016). Plasma Resuscitation Promotes Coagulation Homeostasis Following Shock-Induced Hypercoagulability. Shock.

[11] Fries D., Martini W.Z. (2010). Role of fibrinogen in trauma-induced coagulopathy. Br. J. Anaesth..

[12] Rahbar E., Cardenas J.C., Matijevic N., Del Junco D., Podbielski J., Cohen M.J., Cotton B.A., Holcomb J.B., Wade C.E. (2015). Trauma, Time, and Transfusions: A Longitudinal Analysis of Coagulation Markers in Severely Injured Trauma Patients Receiving Modified Whole Blood or Component Blood Products. Shock.

[13] Berseus O., Boman K., Nessen S.C., Westerberg L.A. (2013). Risks of hemolysis due to anti-A and anti-B caused by the transfusion of blood or blood components containing ABO-incompatible plasma. Transfusion.

[14] Woolley T., Thompson P., Kirkman E., Reed R., Ausset S., Beckett A., Bjerkvig C., Cap A.P., Coats T., Cohen M. (2018). Trauma Hemostasis and Oxygenation Research Network position paper on the role of hypotensive resuscitation as part of remote damage control resuscitation. J. Trauma Acute Care Surg..

[15] Moore E.E., Thomas G. (1996). Orr Memorial Lecture. Staged laparotomy for the hypothermia, acidosis, and coagulopathy syndrome. Am. J. Surg..

[16] Tisherman S.A., Stein D.M. (2018). ICU Management of Trauma Patients. Crit. Care Med..

[17] Harvin J.A., Mims M.M., Duchesne J.C., Cox C.S., Wade C.E., Holcomb J.B., Cotton B.A. (2013). Chasing 100%: The use of hypertonic saline to improve early, primary fascial closure after damage control laparotomy. J. Trauma Acute Care Surg..

[18] Mira J.C., Brakenridge S.C., Moldawer L.L., Moore F.A. (2017). Persistent Inflammation, Immunosuppression and Catabolism Syndrome. Crit. Care Clin..

